# Adeno-associated virus vectored immunoprophylaxis to prevent HIV in healthy adults: a phase 1 randomised controlled trial

**DOI:** 10.1016/S2352-3018(19)30003-7

**Published:** 2019-03-15

**Authors:** Frances H Priddy, David J M Lewis, Huub C Gelderblom, Hana Hassanin, Claire Streatfield, Celia LaBranche, Jonathan Hare, Josephine H Cox, Len Dally, Daryl Bendel, David Montefiori, Eddy Sayeed, Jim Ackland, Jill Gilmour, Bruce C Schnepp, J Fraser Wright, Philip Johnson

**Affiliations:** a International AIDS Vaccine Initiative, New York, NY, USA; b NIHR Imperial Clinical Research Facility, Imperial College, London UK; c International AIDS Vaccine Initiative, London, UK; d The Emmes Company, LLC, Rockville, MD, USA; e Surrey Clinical Research Centre, University of Surrey, Guildford, UK; f Global Biosolutions, Melbourne, Australia; g Department of Surgery, Duke University Medical Center, Durham, NC, USA; h Children's Hospital of Philadelphia

## Abstract

**Background:**

A preventive vaccine for HIV is a crucial public health need; adeno-associated virus (AAV)-mediated antibody gene delivery could be an alternative to immunisation to induce sustained expression of neutralising antibodies to prevent HIV. We assessed safety and tolerability of rAAV1-PG9DP, a recombinant AAV1 vector encoding the gene for PG9, a broadly neutralising antibody against HIV.

**Methods:**

This first-in-human, proof-of-concept, double-blind, phase 1, randomised, placebo-controlled, dose-escalation trial was done at one clinical research centre in the UK. Healthy men aged 18–45 years without HIV infection were randomly assigned to receive intramuscular injection with rAAV1-PG9DP or placebo in the deltoid or quadriceps in one of four dose-escalating cohorts (group A, 4 × 10^12^ vector genomes; group B, 4 × 10^13^ vector genomes; group C, 8 × 10^13^ vector genomes; and group D, 1·2 × 10^14^ vector genomes). Volunteers were followed up for 48 weeks. The primary objective was to assess safety and tolerability. A secondary objective was to assess PG9 expression in serum and related HIV neutralisation activity. All volunteers were included in primary and safety analyses. The trial is complete and is registered with ClinicalTrials.gov, number NCT01937455.

**Findings:**

Between Jan 30, 2014, and Feb 28, 2017, 111 volunteers were screened for eligibility. 21 volunteers were eligible and provided consent, and all 21 completed 48 weeks of follow-up. Reactogenicity was generally mild or moderate and resolved without intervention. No probably or definitely related adverse events or serious adverse events were recorded. We detected PG9 by HIV neutralisation in the serum of four volunteers, and by RT-PCR in muscle biopsy samples from four volunteers. We did not detect PG9 by ELISA in serum. PG9 anti-drug antibody was present in ten volunteers in the higher dose groups. Both anti-AAV1 antibodies and AAV1-specific T-cell responses were detected.

**Interpretation:**

Future studies should explore higher doses of AAV, alternative AAV serotypes and gene expression cassettes, or other broadly neutralising HIV antibodies.

**Funding:**

International AIDS Vaccine Initiative, United States Agency for International Development, Bill & Melinda Gates Foundation, US National Institutes of Health.

## Introduction

An effective HIV vaccine is needed to end the HIV epidemic. Although traditional immunisation approaches for HIV have not been successful, significant advances have been made in identifying and characterising naturally occurring human antibodies against HIV that are both broad and potent and capable of neutralising diverse isolates of HIV at very low concentrations.^1^, ^2^ Multiple broadly neutralising HIV antibodies are under development for use as immunoprophylaxis against HIV.^3^ However, even with modifications to prolong the antibody half-life, this modality will require repeated administration to maintain protection and might not be as cost-effective or acceptable as a vaccine. Adeno-associated virus (AAV)-mediated expression of HIV antibodies could be an alternative to immunisation, if sufficient concentrations of neutralising antibodies can be sustained after a single AAV administration.

This approach draws on the gene therapy field in which AAV vectors encoding sequences of deficient proteins are given by various routes to induce sustained expression and provide functional improvement or prevention of progression.^4^, ^5^ For example, intramuscular administration of a recombinant AAV (rAAV) vector encoding the alpha-1 antitrypsin (AAT; also known as serpin family A member 1) gene in patients with AAT deficiency induced sustained protein expression for more than 5 years.^6^ AAV vectors have advantages over other platforms for gene delivery. Although most humans are naturally exposed to wild-type AAV, it is not pathogenic. rAAV vectors can accept large gene sequence inserts and can infect a variety of tissues but do not have the viral elements necessary for replication, even in the presence of helper viruses. Persistence of rAAV-based gene sequences in cells are long lived and do not commonly involve integration into nuclear DNA.^7^

**Research in context**

**Evidence before this study**

We searched PubMed from Sept 8, 2012, to July 3, 2013, for all clinical studies on adeno-associated virus (AAV) gene therapy or vectored immunoprophylaxis in English with no publication date restrictions. We searched ClinicalTrials.gov during the same period for all completed and ongoing AAV or gene therapy trials. Search terms for both searches were ”AAV”, ”gene therapy”, ”clinical trials”, and ”vectored immunoprophylaxis”, alone and in combinations. There were no ongoing or completed studies of vectored immunoprophylaxis or using AAV gene delivery for prevention of infectious disease or delivery of antibodies in healthy people. One non-human primate study showed proof-of-concept for AAV1-delivered antibody-like genes to protect against chimeric simian-HIV challenge. Numerous animal models and phase 1 and 2 clinical trials assessed AAV-based gene therapy using various AAV serotypes, doses, and routes of administration for treatment of inherited and acquired diseases and had potential benefit but with variable clinical effect. Two clinical trials showed that AAV1 gene therapy given intramuscularly at doses of 10^14^ vector genomes could induce protein expression up to 36 μg/mL for at least 1 year.

**Added value of this study**

To our knowledge, this is the first trial using gene therapy techniques for prevention of disease and for antibody delivery. The study demonstrates that neutralising responses to HIV can be induced using vectored immunoprophylaxis with AAV, and this approach might be feasible for infectious disease prevention. The presence of durable anti-drug antibody responses indicates there was HIV antibody expression. Our data suggests that anti-drug antibody responses might have diminished HIV antibody expression. Different HIV antibodies and different AAV serotypes might improve transduction and expression after intramuscular administration. This data contributes to the safety profile of AAV use in healthy populations. We provide evidence that AAV doses up to 1·2 × 10^14^ vector genomes given intramuscularly are safe in healthy adults.

**Implications of all the available evidence**

Vectored immunoprophylaxis is a novel approach for prevention of infectious diseases of public health significance, such as HIV, for which traditional vaccines do not exist. We show that AAV-based prevention for HIV might be safe and feasible for healthy populations. However, technical improvements in this approach are now needed to increase vector transduction and antibody expression, and to reduce anti-vector and anti-antibody immune responses. This study provides a foundation for a novel prevention approach that might be applied to other infectious diseases of global relevance.

The potential for rAAV-vectored gene delivery for infectious disease prevention, termed vectored immunoprophylaxis, was demonstrated for HIV in a non-human primate model with rAAV serotype 1 expressing a simian immunodeficiency virus (SIV) immunoadhesin.^8^, ^9^ Immunoadhesins are fusion proteins that combine the functional domain of antibodies with immunoglobulin constant domains. After a single injection of rAAV1-SIV immunoadhesin, neutralising activity in serum lasted up to 6 years and completely protected six (67%) of nine animals against intravenous challenge with virulent SIV.^8^ The identification of new HIV neutralising antibodies with increased breadth and potency makes passive immunoprophylaxis theoretically possible for HIV prevention.^10^ Although this strategy is effective for infants at high risk of respiratory syncytial virus infection, wide-scale use of passively injected monoclonal antibodies for adult HIV prevention faces significant resource constraints. Vectored immunoprophylaxis could provide long-lasting production of neutralising antibodies without the cost, acceptability, and implementation hurdles of repeated injections.

rAAV1-PG9DP is a recombinant adeno-associated virus serotype 1 (rAAV1) vector containing gene sequences of PG9. PG9 is a human monoclonal IgG1 antibody that reacts with the V1V2 loop of the HIV-1 envelope gp120 protein and was derived from a patient with clade A HIV infection.^11^ PG9 was selected as the transgene for this prototype product because it is highly potent and has the ability to neutralise a broad panel of HIV strains. The median concentration of PG9 antibody necessary to achieve 90% inhibition of a diverse set of HIV-1 viruses is ten to 60 times lower than previously identified monoclonal antibodies.^11^ The PG9 IgG1 heavy and light chain genes were synthesised using optimal human codon usage for increased expression in a rAAV vector. The amino acid sequence of the codon-optimised PG9 is identical to the original PG9 patient isolate, with the exception of the addition of a 21-amino acid synthetic signal peptide needed for proper secretion into the serum.^12^ The expression cassette consists of a dual promoter system expressing the PG9 variable heavy and variable light domains as an IgG1 antibody (appendix p 2). The heavy and light chains are expressed under the control of a CMV and EF1a promoter, respectively, and self-assemble into the PG9 IgG1.

To assess the concept of vectored immunoprophyaxis for HIV prevention, we assessed safety and tolerability of rAAV1-PG9DP in healthy men.

## Methods

### Study design and participants

This first-in-human, proof-of-concept, double-blind, phase 1, randomised-within-cohort, placebo-controlled, dose-escalation trial was done at a single site, the University of Surrey Clinical Research Centre (Guildford, UK).

We screened healthy men aged 18–45 years. Inclusion criteria were good health as assessed by history, physical examination, and clinical laboratory assessment; ability to understand and comply with the protocol and provide written informed consent; and willingness to have HIV testing and risk reduction counselling, use condoms for 3 months after study injection, and forego blood or other tissue donation during the study. For the fourth cohort, volunteers had to be willing to undergo muscle biopsy at 12 weeks and 48 weeks after injection. Exclusion criteria included HIV-1 or HIV-2 infection, active hepatitis B or hepatitis C infection, pre-existing antibodies to AAV1, previous receipt of another AAV vector, eligible for HIV vaccine, monoclonal antibody or polyclonal immunoglobulin, receipt of a vaccine within 14 days (killed vaccines) or 60 days (live attenuated vaccines), body-mass index (BMI) of 30·0 kg/m^2^ or more, and specific risk behaviour for HIV infection. Women were excluded because of the potential for long-term antibody expression and the theoretical risk to a future pregnancy. Full inclusion and exclusion criteria are given in the protocol, which is available online.

Volunteers provided written informed consent before the start of the study. The protocol was approved by the University of Surrey Ethics Committee, the NHS Research Ethics Service Bristol Research Ethics Committee and the UK Medicines and Healthcare products Regulatory Authority and was done in compliance with Good Clinical Practices and the Declaration of Helsinki guidelines.

### Randomisation and masking

Groups of four or five eligible volunteers were assigned to four dose-escalation cohorts in the order of enrolment and randomly assigned in a 3:1 (first four cohorts) or 4:1 (fifth cohort) ratio to intervention or control groups using a permuted block design. Volunteers and study staff, except for study staff preparing injections, were masked to treatment allocation within a cohort.

### Procedures

Volunteers received intramuscular injection with rAAV1-PG9DP (intervention groups) or placebo (control groups) at a single timepoint and were followed up for 48 weeks.

An independent safety review board assessed safety data from at least 6 weeks after administration in the first and second groups and from at least 8 weeks in the third group to allow each dose escalation. After the third group, the safety review board also considered the level of PG9 expression to establish whether to dose escalate to the fourth and highest dose or to expand the third group. At study conclusion, volunteers were encouraged to enrol in an extended follow-up study. We produced rAAV1-PG9DP by transient transfection in human embryonic kidney (HEK293) cells, with AAV1 capsid elements supplied during transfection, and formulated as a liquid for intramuscular injection. We formulated AAV1-PG9DP at a concentration of 4 × 10^13^ vector genomes per mL in an isotonic buffered saline solution. The placebo, also used as a diluent, was the isotonic buffered saline solution. Both the rAAV1-PG9DP vector and placebo were manufactured by the clinical vector core division of the Center for Cellular and Molecular Therapeutics at the Children's Hospital of Philadelphia (Philadelphia, PA, USA) and supplied as sterile, frozen solutions in single-use polypropylene vials stored at −60°C or lower. We thawed and prepared rAAV1-PG9DP with diluent just before injection to concentrations of 4 × 10^12^, 4 × 10^13^, 8 × 10^13^, or 1·2 × 10^14^ vector genomes. We administered injections by 1·0-inch or 1·5-inch, 22-gauge needle in a single volume of 1 mL in one deltoid muscle (4 × 10^12^ vector genomes for group A and 4 × 10^13^ vector genomes for group B), 1 mL in each deltoid (8 × 10^13^ vector genomes for group C), 1·5 mL administration in each deltoid (1·2 × 10^14^ vector genomes for group D), or 0·5 mL at three sites at least 5 cm apart in each vastus lateralis in the quadriceps (1·2 × 10^14^ vector genomes for group D1, six injections; table 1).

**Table 1:** Study design

	**Number of volunteers, intervention:placebo**	**Intervention dose, vector genomes**	**Volume**	**Location**
A	3:1	4 × 10^12^	1·0 mL	Deltoid
B	3:1	4 × 10^13^	1·0 mL	Deltoid
C	3:1	8 × 10^13^	Two injections of 1·0 mL	Deltoids
D	3:1	1·2 × 10^14^	Two injections of 1·5 mL	Deltoids
D1	4:1	1·2 × 10^14^	Six injections of 0·5 mL	Quadriceps

All cohorts received rAAV1-PG9DP or placebo by intramuscular injection at a single timepoint.

Volunteers were monitored for 4 h after injection and provided local and systemic solicited adverse event data for 7 days after administration. Adverse events were recorded up to week 24 and were graded using the Division of AIDS Table for Grading the Severity of Adult and Pediatric Adverse Events, version 1.0.^13^ Follow-up visits for examination and clinical laboratory assessments were done on days 3 and 7 and weeks 2–8, 10, 12, 16, 20, 24, 36, and 48. In addition to standard haematology, chemistry, and urinalysis, assessment for immune-mediated disease included C-reactive protein and complement C3 and C4. Sera were collected at most visits for PG9 ELISA and at monthly visits for HIV neutralisation, PG9 anti-drug antibody, and anti-AAV1 antibody. Whole blood was collected on days 3 and 7 and weeks 2–4 and 12 for AAV1 vector concentration. Peripheral blood mononuclear cells were collected at weeks 6, 12, and 48 for cellular ELISpot assay. In the five volunteers with quadriceps injections (group D1), we obtained muscle biopsy samples from an injection site at 12 weeks and 48 weeks after injection (appendix p 1).

We assessed PG9 expression in serum with an ELISA assay using anti-PG9 Fab as a capture reagent, with lower limit of quantification of 2·5 μg/mL. We assessed HIV neutralisation in serum samples screened against a panel of nine HIV pseudoviruses (appendix p 4). We assessed for presence of PG9 anti-drug antibody by a three-step algorithm with a Meso-Scale Discovery-electohemiluminescence (MSD-ECL) bridging assay for screening, followed by an MSD-ECL bridging competition assay for confirmation, and an adapted neutralisation assay to assess anti-drug antibody inter ference with PG9 function. The adapted neutralisation assay measured a reduction in the neutralising activity of a 50% inhibitory dose of PG9 against HIV-1 REJO4541.67 Env-pseudotyped virus using a validated TZM-bl cell assay.^14^ Anti-drug antibody was considered positive if the inhibitory activity of PG9 was reduced by at least 50%.

Titre of anti-AAV1 antibodies was assessed in serum samples by ELISA using AAV1 and AAV2 capsid protein as capture reagent. T-cell responses specific for AAV1 capsid protein were established by interferon-γ ELISpot on cryopreserved peripheral blood mononuclear cells, using four peptide pools each containing 45 AAV1 capsid peptides, with a positive response defined as more than 38 spot-forming units per 10^6^ peripheral blood mononuclear cells and mean spot-forming units three times greater than background response.

Persistence of vector DNA in whole blood and muscle was assessed by use of a real-time PCR assay with primers and probes specific to the cytomegalovirus promoter and PG9 antibody gene of rAAV1-PG9DP. Expression of PG9 mRNA in muscle was assessed with a real-time RT-PCR assay with the same PG9 specific primers and probes. Expression of total IgG in muscle was assessed by use of immunohistochemistry.

### Outcomes

The primary objective of this study was to assess the safety and tolerability of rAAV1-PG9DP when administered intramuscularly in healthy men by careful assessment of reactogenicity and safety parameters. The secondary objectives were to assess PG9 expression, the extent and duration of HIV neutralisation activity, the development of anti-PG9 and anti-AAV1 antibodies, and to assess anti-AAV1 T-cell responses and the distribution of AAV1 vector in the blood.

### Statistical analysis

Because this was a phase 1, proof-of-concept study, no statistical estimation was done for sample size. Cohort size of four per group was considered adequate to collect first-in-human safety and expression data, and to select an AAV1 dose for development of next-generation inserts. Summaries of continuous and categorical variables were calculated using SAS software, version 9.3. This trial is registered with ClinicalTrials.gov, number NCT01937455.

### Role of the funding source

The International AIDS Vaccine Initiative (IAVI) designed the trial, monitored the clinical site, oversaw data collection, analysed and interpreted data, and wrote the report. IAVI's donors had no role in study design, data collection, data analysis, data interpretation, or writing of the report. The corresponding author had full access to all the data in the study and had final responsibility for the decision to submit for publication.

## Results

**Figure 1:**
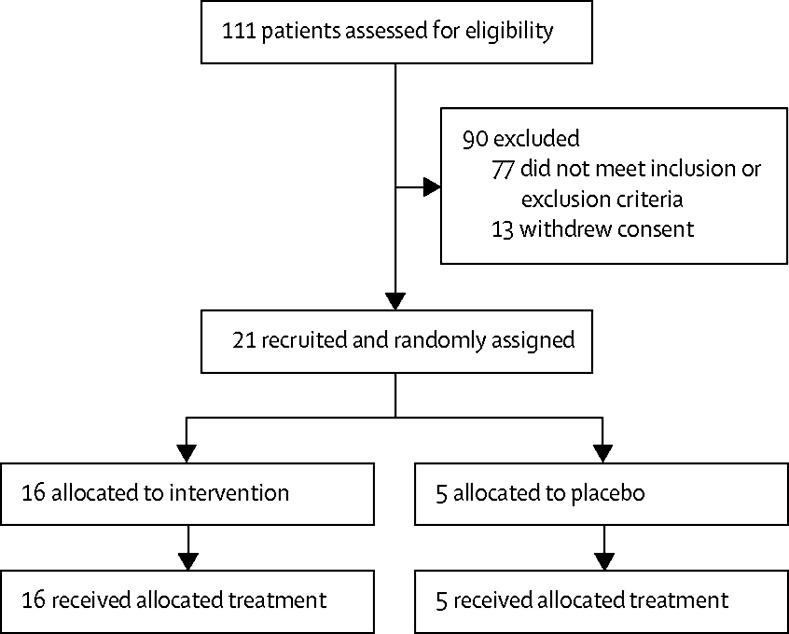
Trial profile

Between Jan 30, 2014, and Feb 28, 2017, 111 volunteers were screened for the study; 77 were ineligible because they did not meet inclusion or exclusion criteria. Of these, 36 were ineligible because of clinically relevant medical history or examination or laboratory finding, 27 because of pre-existing AAV1 or AAV2 antibody, seven because of HIV risk behaviour, four because of high BMI, and three because of not understanding the study. 13 were ineligible because they withdrew consent. 21 volunteers were enrolled and randomly assigned to receive intervention (n=16) or placebo (n=5). All 21 volunteers received intramuscular injection and completed 48 weeks of follow-up (figure 1). By design, all volunteers were men, mean age was 26 years (range 18–41; table 2).

**Table 2:** Baseline characteristics of trial volunteers

		**Group A (n=3)**	**Group B (n=3)**	**Group C (n=3)**	**Group D (n=3)**	**Group D1 (n=4)**	**Placebo (n=5)**	**Total (n=21)**
Race and ethnicity								
	Asian	0	0	0	1 (33%)	0	1 (20%)	2 (10%)
	White and not Hispanic or Latino	3 (100%)	3 (100%)	3 (100%)	2 (67%)	4 (100%)	3 (60%)	18 (86%)
	White and Hispanic or Latino	0	0	0	0	0	1 (20%)	1 (5%)
Mean age, years		24 (21–27)	28 (20–36)	22 (19–27)	29 (19–38)	25 (18–37)	28 (19–41)	26 (18–41)
Mean body-mass index, kg/m^2^		27·4 (21·9–36·8)	23·8 (19·6–28·4)	23·3 (22·0–24·2)	22·4 (21·9–23·2)	22·9 (18·7–26·9)	27·0 (24·3–30·7)	24·6 (18·7–36·8)

Data are n (%) or mean (range).

Safety data were available from all volunteers. Solicited local and systemic adverse events collected in the 7 days after injection were primarily mild and all resolved without intervention. No severe or very severe reactions occurred. Injection site pain and tenderness, myalgia, and headache were the most common reactions (table 3). No serious adverse events and no probably or definitely related unsolicited adverse events occurred. 59 unsolicited adverse events were reported in 20 (95%) of the 21 volunteers between the day of injection (day 0) and week 24 (appendix p 3). Of these, ten occurred within 7 days of injection. One (2%) of the 59 events, in a volunteer in group A, was considered possibly related to injection: mild musculoskeletal pain at the injection site that began within 7 days of injection and resolved without sequelae at 33 days. Adverse events included upper respiratory infections, headaches, musculoskeletal pain, and trauma (appendix p 3). No events or laboratory abnormalities were counted as probably or definitely related to injection or as possible autoimmune disease.

**Table 3:** Volunteers with local and systemic solicited adverse events within 7 days of product administration

	**Group A (n=3)**		**Group B (n=3)**		**Group C (n=3)**		**Group D (n=3)**		**Group D1 (n=4)**		**Placebo (n=5)**	
	Mild	Moderate	Mild	Moderate	Mild	Moderate	Mild	Moderate	Mild	Moderate	Mild	Moderate
Pain	0	0	1 (33%)	1 (33%)	1 (33%)	1 (33%)	1 (33%)	2 (67%)	2 (50%)	1 (25%)	2 (40%)	0
Tenderness	2 (67%)	0	3 (100%)	0	2 (67%)	1 (33%)	1 (33%)	2 (67%)	2 (50%)	0	2 (40%)	0
Chills	0	0	0	0	1 (33%)	0	1 (33%)	0	1 (25%)	0	0	0
Malaise	0	0	0	0	0	0	0	2 (67%)	2 (50%)	1 (25%)	0	0
Myalgia	0	0	0	0	1 (33%)	1 (33%)	1 (33%)	1 (33%)	2 (50%)	1 (25%)	1 (20%)	0
Headache	1 (33%)	0	2 (67%)	0	1 (33%)	0	2 (67%)	1 (33%)	2 (50%)	1 (25%)	2 (40%)	0
Nausea	0	0	0	0	0	0	1 (33%)	0	1 (25%)	0	0	0

Data are n (%). No vomiting, fever, erythema, or induration adverse events were reported.

**Figure 2:**
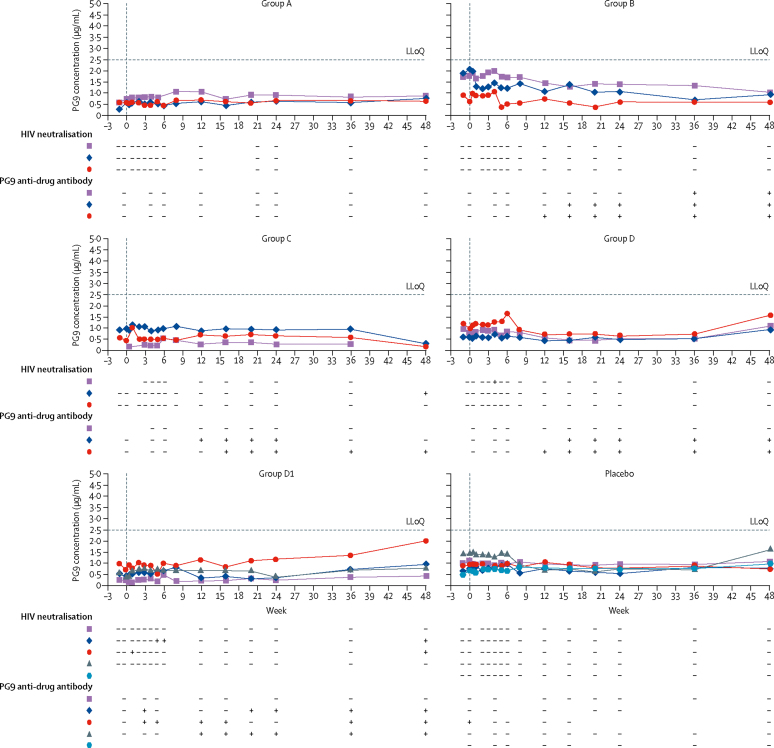
Serum PG9 concentration, HIV neutralisation, and presence of confirmed anti-PG9 antibody Each coloured line corresponds to a different volunteer. – under HIV neutralisation indicates absence and + indicates presence of HIV neutralisation on either a nine-virus pseudovirus panel or to CAP45.2.00.G3 pseudovirus; – under PG9 anti-drug antibody indicates absence and + indicates presence of confirmed tier 2 PG9 anti-drug antibody. Group A=4 × 10^12^ vector genomes. Group B=4 × 10^13^ vector genomes. Group C=8 × 10^13^ vector genomes. Group D and group D1=1·2 × 10^14^ vector genomes. LLoQ=lower limit of quantification.

We did not detect PG9 by quantitative ELISA (lower limit of quantification of 2·5 μg/mL) at any timepoint in any group (figure 2). One volunteer in group C had HIV neutralisation detectable at the final study visit (week 48) against two viruses on a nine-virus HIV pseudovirus panel, with a concentration needed to achieve 50% inhibition (IC50) of 10·2 μg/mL to NL4-3 (a sensitive virus) and IC50 of 11·2 μg/mL to JR-CSF (a modestly resistant virus). One volunteer in group D had HIV neutralisation detectable at week 4 to NL4-3 with an IC50 of 11·8 μg/mL. No HIV neutralisation was observed for the other 19 (90%) volunteers on the nine-virus HIV pseudovirus panel. When tested against an HIV pseudovirus CAP45.2.00.G3, which is known to be extremely sensitive to PG9-mediated neutralisation (dilution at which luminescence is diminished by 50% [ID50] of <0·01 μg/mL), serum samples from two volunteers in group D1 had neutralisation; one at week 6 (ID50 53 μg/mL), week 8 (74 μg/mL), and week 48 (28 μg/mL) and the other at week 2 (25 μg/mL) and week 48 (33 μg/mL; figure 2).

PG9 anti-drug antibodies were confirmed in all three rAAV1-PG9DP recipients in group B, two of three recipients in both groups C and D, and three of four recipients in group D1. These responses were first detectable as early as week 4 and most persisted through to the final study visit (week 48). Of the 10 rAAV1-PG9DP recipients with confirmed anti-drug antibody, five volunteers had functional ability to inhibit PG9-mediated neutralisation in an anti-drug antibody neutralisation assay (>50% neutralisation inhibitory activity).

Anti-drug antibody was detected in a similar proportion of volunteers with HIV neutralisation (three [75%] of four) to those without neutralisation (seven [58%] of 12); results for functional anti-drug antibody were similar (two [50%] of four volunteers with neutralisation *vs* three [25%] of 12 volunteers without neutralisation; appendix p 4).

**Figure 3:**
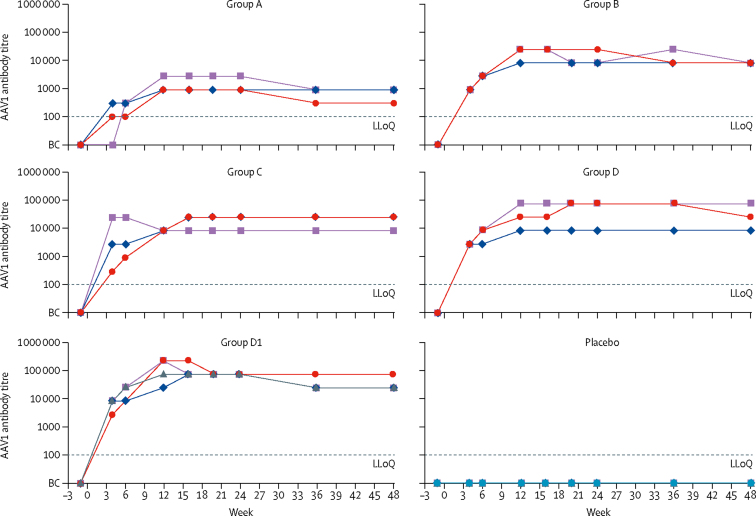
Serum AAV1 antibody titres Each coloured line corresponds to a different volunteer. BC=below cutoff. Group A=4 × 10^12^ vector genomes. Group B=4 × 10^13^ vector genomes. Group C=8 × 10^13^ vector genomes. Group D and group D1=1·2 × 10^14^ vector genomes. LLoQ=lower limit of quantification.

Volunteers in all dose groups who received rAAV1-PG9DP developed detectable anti-AAV1 antibodies by week 4, which persisted throughout the study (figure 3). Based on either the mean response or area under the curve per volunteer from week 4 to their last observation, there seems to be a dose–response relationship with mean anti-AAV1 antibody titres of 1/973 for group A, 1/11 250 for group B, 1/15 125 for group C, 1/33 525 for group D, and 1/60 581 for group D1. Cellular responses to AAV1 capsid, measured by interferon (IFN)-γ ELISpot, were present in one of three rAAV1-PG9DP recipients in group B, two of three recipients in both groups C and D, and in two of four recipients in group D1 (appendix p 4). AAV1 capsid-specific T-cell responses were observed in four volunteers at both weeks 6 and 12 in groups C and D but decreased by week 48. In group D1, magnitude of T-cell responses were similar between weeks 6, 12, and 48.

No association was noted between the number of volunteers with confirmed positive anti-drug antibody or AAV1-specific IFN-γ ELISpot responses and the number with any adverse events, including clinical laboratory markers of hepatic or renal function or inflammation (C-reactive protein and complement C3 and C4).

**Figure 4:**
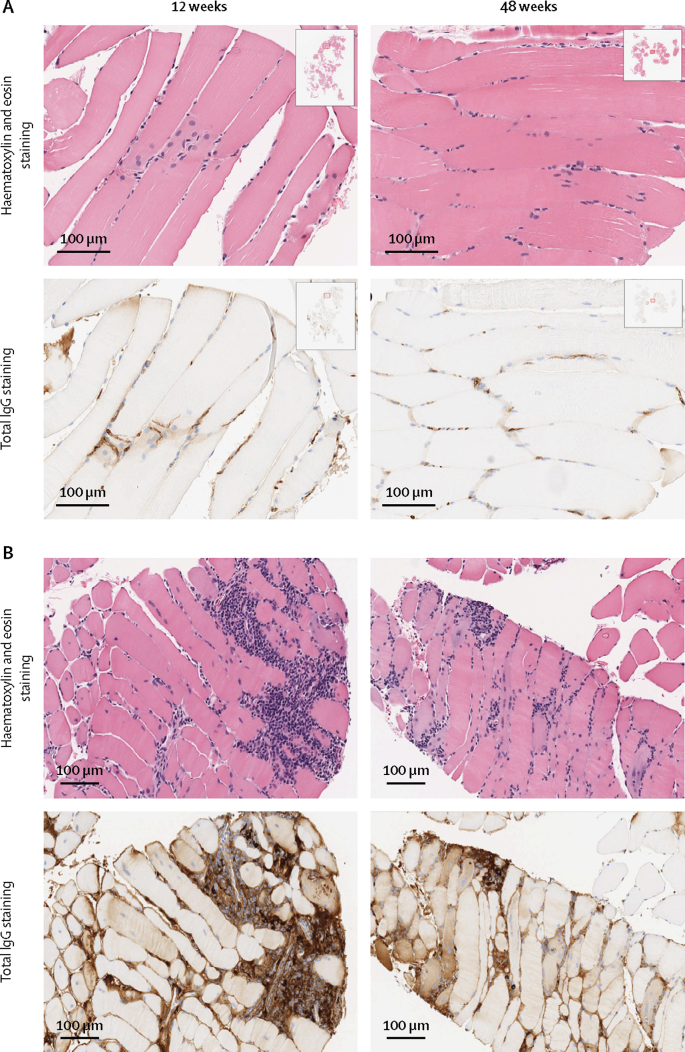
Quadriceps muscle biopsy staining (A) Sections from a placebo recipient. (B) Sections from an AAV1-PG9DP recipient from group D1.

We detected rAAV1-PG9DP vector DNA sequences for both the promoter and the PG9 gene in whole blood in all 13 volunteers who received the product in groups B, C, D, and D1 until week 12, and in two of three volunteers in group A until day 7 and week 4 (appendix p 4). In group D1, in muscle biopsy samples at 12 weeks and 48 weeks after injection, we detected PG9 mRNA by PCR in all four volunteers who received rAAV1-PG9DP. These volunteers also had increased total IgG by histochemistry both intracellularly and in extracellular spaces at 12 weeks (four volunteers) and at 48 weeks (three volunteers; figure 4). In the muscle biopsy samples, we observed a consistent pattern of lymphocytic infiltration in areas of IgG-positive myocytes in volunteers in group D1, demonstrated by increased purple nuclear elements with haematoxylin and eosin staining and brown shaded myofibres and brown staining around the extracellular lymphocytic infiltrate with total IgG staining, compared with normal tissues taken from volunteers who received placebo (figure 4).

## Discussion

Vectored immunoprophylaxis to prevent HIV seems to be safe in a small number of healthy volunteers, and it induced serum neutralisation in four volunteers, intermittently for at least a year. Muscle biopsy sample testing confirmed the presence of PG9 secreted from cells. However, antibody expression was too low to be directly detected in the circulation. To our knowledge, this is the first use of AAV vectors in healthy populations for antibody gene delivery for an infectious disease prevention indication. rAAV1-PG9DP was well tolerated. Solicted adverse events were generally mild or moderate and resolved without intervention. No probably or definitely related adverse events and no serious adverse events occurred. These findings are consistent with or milder than the expected safety profile of a preventive vaccine. Notably, despite the presence of immune responses to both the AAV vector and the PG9 antibody, we did not detect evidence of immune-mediated disease with frequent safety assessments, including specific end-organ assessment and testing for general markers of inflammation such as C-reactive protein, over a 1-year period. In some therapeutic applications of AAV, inflammatory hepatic responses have been noted after intravenous AAV administration, which appear to be suppressed with oral steroids.^15^, ^16^, ^17^ Our findings, although in only a few volunteers, suggest that potential safety concerns such as increases in hepatic transaminase concentrations noted with intravenous AAV administration or antibody-induced autoimmune disease might not be applicable to this approach using intramuscular AAV administration and a fully human antibody transgene. To be feasible as a universal preventive intervention in healthy populations, vectored immunoprophylaxis for HIV should have a safety profile similar to licensed vaccines.

Several factors might have led to lower than expected transgene expression, including AAV vector genome dose, AAV capsid serotype, transgene expression promoter design, the antibody itself, or abrogating cellular or humoral responses to the vector or antibody. We selected intramuscular administration as the most feasible route for an injectable preventive HIV candidate in healthy populations. The target vector genome dose of 10^13^ to 10^14^ was established from existing safety and expression data from the gene therapy field, as well as manufacturing yields that limited vector genome concentration to 4 × 10^13^ vector genomes per mL. A phase 2 clinical trial by Flotte and colleagues^18^ showed successful AAT protein expression after intramuscular administration of recombinant AAV1 with a linear dose response. Increased AAT expression was detected at doses of approximately 4·2 × 10^13^ vector genomes up to 4·3 × 10^14^ vector genomes, with peak expression in the highest dose of 21–36 μg/mL.

Data from a preclinical study done by our group in immunocompromised Rag1 mice, administration of rAAV1-PG9DP at 3 × 10^11^ vector genomes resulted in PG9 expression of 53 μg/mL (unpublished). In humans, we expected detectable PG9 (>2·5 μg/mL) in groups C (8 × 10^13^ vector genomes) and D (1·2 × 10^14^ vector genomes). rAAV1-PG9DP was administered with a single injection in one or both deltoid muscles in groups A–D, whereas the rAAV1-AAT product was split in up to 100 intramuscular injections over ten different muscle sites. Although the absolute vector genomes delivered in the two studies were similar, rAAV1-PG9DP had significantly less exposure to muscle tissue, which might have limited uptake and expression. In our study, only groups with the highest dose showed evidence of neutralising activity. Only group D1, which received six injections over two larger quadriceps muscles, had neutralisation of a virus highly sensitive to PG9, suggesting that broader muscle tissue exposure increases transgene expression. Administration of larger vector genome doses at multiple muscle locations might be needed for adequate antibody expression, but this product profile would be less acceptable for widespread preventive use. Methods for regional muscular administration are being assessed in preclinical studies.

Qualities of PG9 antibody itself might have limited the transgene expression or resulted in immune clearance of expressed PG9. rAAV1-PG9DP was designed to express fully human IgG1 PG9. The observation of IgG antibody staining inside myocytes and in interstitial spaces and of PG9-specific mRNA detection in the muscle biopsy samples provide strong indications that transgene expression was successful and some level of IgG antibody assembly occurred. This observation, coupled with the detection of PG9 anti-drug antibody but without measurable PG9 antibody in serum suggests either that expression was too low to be detected systemically or that PG9 was cleared by the immune system. The structure of PG9 has an unusually long complementary determining region and is sulphated, which might have made it an immune target, despite being fully human. Alternatively, the form of PG9 expressed from the transgene might have had a different structure, making it immunogenic. A primate study showed that PG9 had a two-times shorter plasma half-life and lower protective efficacy than the VRC01 and 10E8 antibodies, which might be related to PG9 binding to host cell glycoproteins.^19^ The high frequency of detection of PG9 anti-drug antibody and the lymphocytic infiltration in muscle biopsy samples in our trial support an immune clearance hypothesis.

In our study, low-level PG9 expression was suggested by detection of HIV neutralisation in four volunteers against laboratory pseudovirus isolates known to be extremely sensitive to PG9 (CAP45·2.00.G3 in two volunteers), somewhat sensitive (NL43 in two volunteers), and somewhat resistant (JRCSF in one volunteer). Anti-drug antibody responses did not appear to be more common in volunteers without neutralisation. However there was a trend for lower frequency of neutralisation in volunteers with functional anti-drug antibody.

Cellular immune responses might also have limited PG9 expression. We detected AAV1 capsid-specific CD8 responses in seven (44%) of 16 recipients, more at higher doses, between weeks 7 and 12. Although low in magnitude, these responses might have contributed to clearing AAV1-transduced cells during the time when antibody expression would be expected to be detectable. Six (86%) of seven volunteers with AAV1-specific ELISpot responses also had confirmed anti-drug antibody responses. These volunteers might have had early, low-level PG9 expression that was sufficient to induce anti-drug antibody, but never reached detectable levels because of T-cell-mediated clearance of AAV1-transduced cells. Clinically significant AAV capsid-specific T-cell responses were detected in a few gene therapy studies after intravenous AAV administration directed to the liver, which correlated with a reduction in transgene expression.^15^, ^20^ These responses were associated with increases in hepatic transaminase concentrations and appeared to be abrogated with oral prednisone. In our trial, AAV1-specific T-cell responses were of low magnitude and not associated with laboratory abnormalities, suggesting cellular clearance. We hoped intramuscular administration would minimise immune clearance of AAV-infected cells. In trials of AAV1-AAT given intramuscularly, anti-AAT antibodies were not detected and cellular responses to the vector were not associated with increases in transaminase concentrations or decreases in transgene expression.^18^, ^21^, ^22^

We selected the AAV1 serotype because previous preclinical and clinical studies have been done with AAV1 vectors for intramuscular delivery. AAV1 showed significantly higher transgene expression than other serotypes in skeletal muscle of multiple animal species.^23^, ^24^ Other studies suggest that AAV8 has similar transduction efficiency.^25^ Muscle biopsy samples obtained 12 weeks and 48 weeks after rAAV1-AAT injection contained rAAV vector genomes, and molecular clones of vector genomes derived from the samples were transcriptionally active.^26^ In our trial, intramuscular administration of rAAV1-PG9DP to volunteers without pre-existing AAV1 immunity resulted in 100% seroconversion, suggesting that the vector successfully transduced skeletal muscle cells. However, it might have extravasated to the peripheral circulation or been taken up by other cell types. The prompt induction of anti-AAV antibody responses might limit the potential for repeat vector administration.

Promoter selection might also affect transgene expression. In the dual promoter design used here, heavy and light chains are expressed from two different promoters with no need for a cleavage signal. We compared this to a single promoter strategy, in which a 24-amino acid, non-self cleavage sequence is used to separate PG9 heavy and light chains after protein synthesis. In vitro, cells transfected with either construct showed neutralisation potency and breadth on a standard panel of HIV isolates similar to native PG9. Both constructs induced similar levels of PG9 expression in immunodeficient mice sera (IAVI, unpublished). The dual promoter was selected to avoid a non-self component with possible immunogenicity effects. However, the dual promoter might create an imbalance between heavy and light chain production because of differential activity of the promoters.

Our study has several limitations, including the small number of volunteers. Additionally, we could not achieve larger vector genome delivery because of the low concentration of the product and desire to avoid multiple intramuscular injections. We were also unable to directly quantify systemic PG9 at concentrations of less than 2·5 μg/mL. HIV antibodies with 10–100-times greater potency than PG9 have now been characterised, with median inhibitory concentrations (IC50) of less than 0·01 μg/mL against sensitive viruses and protection against challenges with simian human immunodeficiency virus (SHIV) at serum concentrations of less than 0·75 μg/mL.^27^, ^28^ An AAV8-based construct containing the VRC07 HIV antibody, which has a median IC50 against sensitive viruses of less than 1 μg/mL, is in phase 1 testing.^29^ Lower concentrations of these HIV antibodies might be biologically relevant for prevention and future studies might need to validate lower limits of quantification for antibody concentration. In-vitro studies and modelling suggest that two or more HIV broadly neutralising antibodies might be needed to protect against the diversity of HIV strains circulating globally.^30^ Muscle biopsy samples could not be adequately tested for PG9 by ELISA because of low availability of samples; furthermore, the samples were received in storage buffers tailored for PCR and immunohistochemistry Furthermore, the in-vivo neutralisation activity detected in a small number of volunteers was close to the assay cutoff and might have been non-specific.

In summary, intramuscular administration of rAAV1-PG9DP in a small number of healthy men appeared safe and well tolerated. PG9 mRNA was detected at the injection site but PG9 antibody was only indirectly detected in serum and was not quantifiable, suggesting very low expression. Our data suggests that some combination of vector serotype, characteristics of the antibody, and host immune response was responsible for low antibody expression. This first-in-human study of the concept of vectored immunoprophylaxis for infectious diseases suggests that improvements are needed to make it a viable method for long-term protective antibody expression; future studies should explore higher doses of AAV, alternative AAV serotypes and gene expression cassettes, and other broadly neutralising HIV antibodies.

## Data sharing

Anonymised data is available upon reasonable request to the corresponding author.
