# Correction to *Lancet HIV* 2019; 6: e230–39

**DOI:** 10.1016/S2352-3018(19)30107-9

**Published:** 2019-04-04

*Priddy FH, Lewis DJM, Gelderblom HC, et al. Adeno-associated virus vectored immunoprophylaxis to prevent HIV in healthy adults: a phase 1 randomised controlled trial.* Lancet HIV *2019; **6:** e230–39*—In the Results text, the neutralisation of CAP45.2.00.G3 has been corrected to ID50 titres in reciprocal dilution, not concentrations in μg/mL. This correction was made on April 3, 2019.

