# Supplementary material for: Adeno-associated virus vectored immunoprophylaxis to prevent HIV in healthy adults: a phase 1 randomised controlled trial
Source: Lancet HIV. 2019 Mar 15;6(4):e230–9. doi: 10.1016/S2352-3018(19)30003-7 (PMC6443625; doi:10.1016/S2352-3018(19)30003-7)
Supplement: Supplementary appendix [file mmc1.pdf]

THE LANCET
HIV

Supplementary appendix

This appendix formed part of the original submission and has been peer reviewed. We post it as supplied by the authors.

Supplement to: Priddy FH, Lewis DJM, Gelderblom HC, et al. Adeno-associated virus vectored immunoprophylaxis to prevent HIV in healthy adults: a phase 1 randomised controlled trial. *Lancet HIV* 2019; published online March 15. http://dx.doi.org/10.1016/S2352-3018(19)30003-7.

Supplementary methods

SRB review after completion of enrollment for each cohort

Briefly, for each dose escalation, blinded interim data 42 days (Groups A & B) or 56 days (Groups C & D) after IMP administration of all volunteers in the respective cohorts was summarized in a report and reviewed by the SRB prior to dose escalation (Groups A, B, C, D). At each SRB review, the safety criteria for dose escalation were met. Dose escalation from Group C to D was also guided by PG9 serum levels, which were negative or well-below the 150ug/mL criteria to stop dose escalation. Cohort expansion at the same dose level from Group D to D1 was guided by dose escalation criteria.

Muscle biopsies

Muscle biopsies of the injection site three and 12 months after IP administration were performed with a disposable 18G TruCore II Biopsy Automatic Biopsy Instrument (Argon Medical, UK) needle to obtain six approximately 20x1x1 mm specimens. Two specimens were immediately frozen in OCT Tissue-Tek in a cryomold (Sakura, Japan). Two specimens were immersed in RNA*later* buffer (ThermoFisher, UK) and stored immediately at 4°C for at least 12 hours but no more than 24 hours, and thereafter stored at –80°C until processing. Two specimens were fixated in 10% neutral buffered formalin (ThermoFisher, UK) for four to 24 hours and then transferred to phosphate buffered saline (PBS, Sigma, UK) until processing.

Immunohistochemistry

For histopathology and immunohistochemistry, paraffin serial sections (4 µm) were stained with hematoxylin and eosin (H&E), and by immunohistochemistry for total IgG with peroxidase conjugated goat anti-human IgG visualized using the Envision FLEX kit (Agilent DAKO).

PG9 mRNA detection

For detection of PG9 mRNA, RNA was isolated from biopsies preserved in RNA*later* using RNeasy mini kit extraction method (QIAgen). We performed real-time RT-PCR using the High-Capacity cDNA kit and TaqMan FAST Advanced Master Mix (ThermoFisher) and primers (Integrated DNA Technologies) and TaqMan probes (Roche) specific for the PG9 gene, using the Applied Biosystems™ QuantStudio™ 7 Flex Real-Time PCR System (ThermoFisher). We used ß-actin as a relative reference standard.

rAAV1-PG9DP vector DNA detection

For detection of rAAV1-PG9DP vector DNA, we isolated DNA from frozen muscle biopsy tissue using the QIAamp DNA mini kit (QIAgen). We performed real-time DNA PCR using the TaqMan FAST Advanced Master Mix (ThermoFisher) and primers (Integrated DNA Technologies) and TaqMan probes (Roche) specific for the CMV promotor and PG9 gene, using the Applied Biosystems™ QuantStudio™ 7 Flex Real-Time PCR System (ThermoFisher). We used a plasmid expressing the rAAV1-PG9DP construct as a reference standard. The CMV promotor primers and probe were designed to not detect wild-type CMV sequences.

Supplementary Figure S1: rAAV1-PG9DP construct

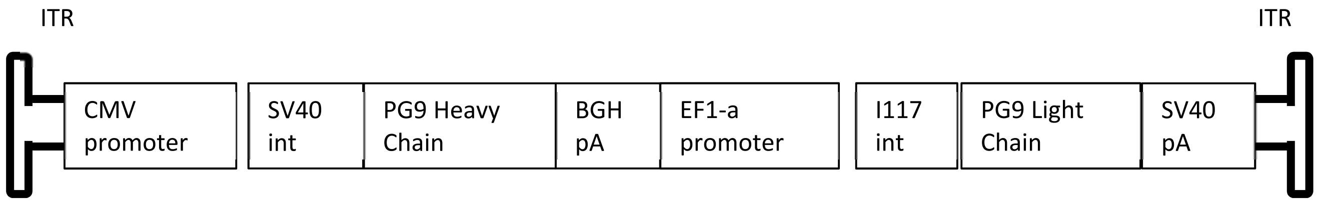

Supplementary Table S1: Any Unsolicited Adverse Event Within 168 Days of Injection

Based on maximum severity per volunteer for each event.

	% Volunteers With Any Event		Number of Volunteers per Dose Group With Any Event					
**Preferred Term**	**Any Active Dose (n=16)**	**Placebo (n=5)**	**Placebo (n=5)**	**Group A 4×10^{12} (n=3)**	**Group B 4×10^{13} (n=3)**	**Group C 8×10^{12} (n=3)**	**Group D $1{\cdot}2 \times 10^{14}$ (n=3)**	**Group D1 $1{\cdot}2 \times 10^{14}$ (n=4)**
Any Event	100·0%	80·0%	4	3	3	3	3	4
Nasopharyngitis	43·8%	20·0%	1[1]	0	1[2]	2[1]	1[1]	3[1, 2]
Musculoskeletal pain	18·9%	20·0%	1[1]	1[1]	0	1[1]	0	0
Gastroenteritis	18·8%	0·0%	0	0	0	2[1, 2]	0	1[1]
Headache	18·8%	20·0%	1[2]	2[1, 2]	0	0	1[1]	0
Sunburn	18·8%	0·0%	0	0	2[1]	1[1]	0	0
Contusion	12·5%	0·0%	0	1[1]	0	0	0	1[1]
Haematuria	12·5%	0·0%	0	0	0	0	1[2]	1[2]
Adjustment disorder with depressed mood	6·3%	0·0%	0	0	1[2]	0	0	0
Atrioventricular block first degree	6·3%	0·0%	0	1[1]	0	0	0	0
Excessive cerumen production	6·3%	0·0%	0	0	1[2]	0	0	0
Haemorrhoids	6·3%	0·0%	0	0	0	0	0	1[1]
Influenza like illness	6·3%	0·0%	0	0	0	0	0	1[2]
Laceration	6·3%	0·0%	0	0	0	0	1[2]	0
Limb injury	6·3%	40·0%	2[1]	0	0	0	1[1]	0
Mass	6·3%	0·0%	0	0	0	0	0	1[1]
Musculoskeletal injury	6·3%	0·0%	0	1[2]	0	0	0	0
Procedural pain	6·3%	0·0%	0	0	0	0	0	1[1]
Rash generalised	6·3%	0·0%	0	0	0	0	0	1[2]
Sleep disorder	6·3%	0·0%	0	1[1]	0	0	0	0
Tooth infection	6·3%	0·0%	0	0	0	1[2]	0	0
Troponin I increased	6·3%	0·0%	0	0	1[4]	0	0	0
Upper respiratory tract infection	6·3%	60·0%	3[1, 2]	0	0	1[1]	0	0
Upper-airway cough syndrome	6·3%	0·0%	0	0	0	0	0	1[1]
Dental discomfort	0·0%	20·0%	1[2]	0	0	0	0	0
Facial bones fracture	0·0%	20·0%	1[2]	0	0	0	0	0
Hypoaesthesia	0·0%	20·0%	1[1]	0	0	0	0	0
Lip dry	0·0%	20·0%	1[1]	0	0	0	0	0
Lower respiratory tract infection viral	0·0%	20·0%	1[1]	0	0	0	0	0
Odynophagia	0·0%	20·0%	1[1]	0	0	0	0	0

Severity of each single event is indicated by: [1] Mild, [2] Moderate, [4] Potentially Life-Threatening.
Fisher's exact 1-tail test of more events in the vaccine groups than placebo (i.e., 16/16 vs 4/5)

Supplementary Table S2: Immunology results by group

Group	ID	PG9 ELISA F(ab)	HIV neut panel	HIV neut CAP45	PG9 ADA	rAAV1-PG9DP vector DNA	Anti-AAV1 ELISA	Anti-AAV1 ELISPOT	rAAV1-PG9DP vector DNA	PG9 mRNA	Total IgG staining
		serum	serum	serum	serum	whole blood	serum	PBMCs	muscle	muscle	muscle
A $4x10^{12}$ vg	039	–	–	–	–	+	+	–			
	004	–	–	–	–	+	+	–			
	001	–	–	–	–	–	+	–			
B $4x10^{13}$ vg	077	–	–	–	+	+	+	–			
	040	–	–	–	+	+	+	–			
	069	–	–	–	+	+	+	+			
C $8x10^{13}$ vg	066	–	–	–	–	+	+	–			
	017	–	+	–	+*	+	+	+			
	060	–	–	–	+*	+	+	+			
D $1{\cdot}2x10^{14}$ vg	064	–	+	–	–	+	+	+			
	089	–	–	–	+	+	+	–			
	035	–	–	–	+*	+	+	+			
D1 $1{\cdot}2x10^{14}$ vg	015	–	–	–	–	+	+	–	+	+	+
	076	–	–	+	+	+	+	+	+	+	+
	034	–	–	+	+*	+	+	–	+	+	+
	084	–	–	–	+*	+	+	+	+	+	+
Placebo	026	–	–	–	–	–	–	–			
	022	–	–	–	–	–	–	–			
	052	–	–	–	–	–	–	–			
	086	–	–	–	–	–	–	–			
	044	–	–	–	–	–	–	–			

Cells show any positive response over all data collected at scheduled post-injection visits. HIV neutralization panel is composed of 9 HIV pseudoviruses (92BR020, 92TH021, 93IN905, 94UG103, MGRM-C-026, MGRM-A-009, MGRM-C-019, JRCSF, NL43). CAP45.2.00.G3 is an HIV virus highly sensitive to PG9 with ID50 <0.01 µg/mL (dilution at which luminescence is diminished 50%).

* Also positive in Tier 3 ADA assay, functional ADA.

Supplementary Figure S2: Serum neutralization titers against CAP45.2.00.G3 for Group D1

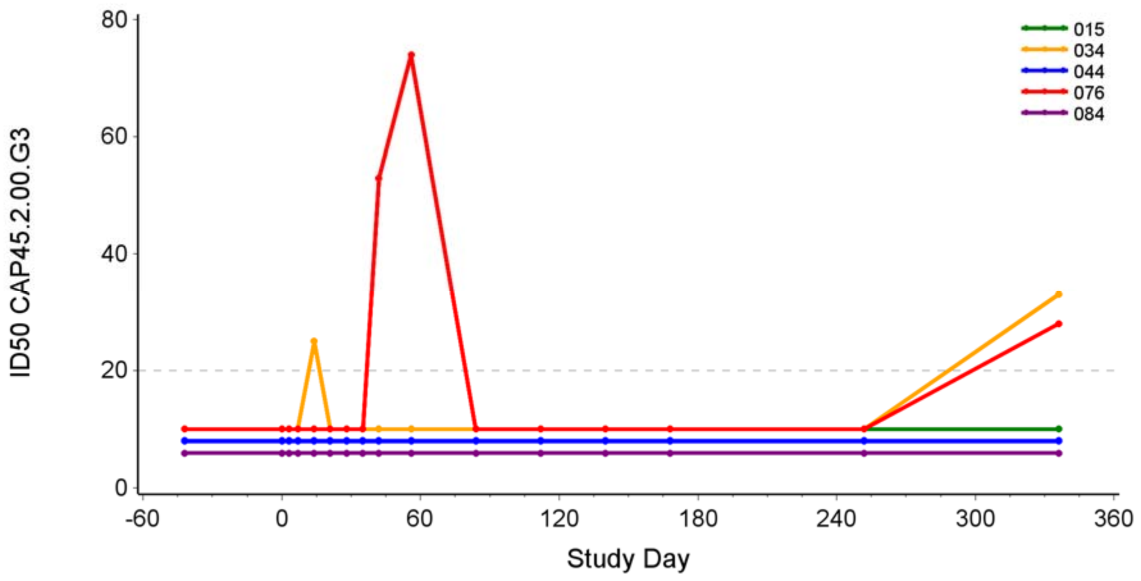

Legend: Colored lines correspond to individual participants in Group D1. Participant 044 received placebo. ID50 is the serum dilution at which luminescence in a luciferase neutralization assay is diminished 50%.

CONSORT 2010 checklist of information to include when reporting a randomised trial*

Section/Topic	Item No	Checklist item	Reported on page No
**Title and abstract**			
	1a	Identification as a randomised trial in the title	1
	1b	Structured summary of trial design, methods, results, and conclusions (for specific guidance see CONSORT for abstracts)	3
**Introduction**			
Background and objectives	2a	Scientific background and explanation of rationale	4
	2b	Specific objectives or hypotheses	9 (Outcomes)
**Methods**			
Trial design	3a	Description of trial design (such as parallel, factorial) including allocation ratio	5
	3b	Important changes to methods after trial commencement (such as eligibility criteria), with reasons	5
Participants	4a	Eligibility criteria for participants	6
	4b	Settings and locations where the data were collected	5
Interventions	5	The interventions for each group with sufficient details to allow replication, including how and when they were actually administered	7
Outcomes	6a	Completely defined pre-specified primary and secondary outcome measures, including how and when they were assessed	9, 7
	6b	Any changes to trial outcomes after the trial commenced, with reasons	NA
Sample size	7a	How sample size was determined	9
	7b	When applicable, explanation of any interim analyses and stopping guidelines	NA
Randomisation:			
Sequence generation	8a	Method used to generate the random allocation sequence	6
	8b	Type of randomisation; details of any restriction (such as blocking and block size)	6
Allocation concealment mechanism	9	Mechanism used to implement the random allocation sequence (such as sequentially numbered containers), describing any steps taken to conceal the sequence until interventions were assigned	6
Implementation	10	Who generated the random allocation sequence, who enrolled participants, and who assigned participants to interventions	6
Blinding	11a	If done, who was blinded after assignment to interventions (for example, participants, care providers, those	6

		assessing outcomes) and how	
	11b	If relevant, description of the similarity of interventions	NA
Statistical methods	12a	Statistical methods used to compare groups for primary and secondary outcomes	9
	12b	Methods for additional analyses, such as subgroup analyses and adjusted analyses	NA
**Results**			
Participant flow (a diagram is strongly recommended)	13a	For each group, the numbers of participants who were randomly assigned, received intended treatment, and were analysed for the primary outcome	10
	13b	For each group, losses and exclusions after randomisation, together with reasons	16
Recruitment	14a	Dates defining the periods of recruitment and follow-up	16
	14b	Why the trial ended or was stopped	12-15
Baseline data	15	A table showing baseline demographic and clinical characteristics for each group	Table 1
Numbers analysed	16	For each group, number of participants (denominator) included in each analysis and whether the analysis was by original assigned groups	10-12, Table 2, Supp Table 1, Supp Table 2
Outcomes and estimation	17a	For each primary and secondary outcome, results for each group, and the estimated effect size and its precision (such as 95% confidence interval)	See 6
	17b	For binary outcomes, presentation of both absolute and relative effect sizes is recommended	See 16
Ancillary analyses	18	Results of any other analyses performed, including subgroup analyses and adjusted analyses, distinguishing pre-specified from exploratory	NA
Harms	19	All important harms or unintended effects in each group (for specific guidance see CONSORT for harms)	10
**Discussion**			
Limitations	20	Trial limitations, addressing sources of potential bias, imprecision, and, if relevant, multiplicity of analyses	16
Generalisability	21	Generalisability (external validity, applicability) of the trial findings	16
Interpretation	22	Interpretation consistent with results, balancing benefits and harms, and considering other relevant evidence	12-15
**Other information**			
Registration	23	Registration number and name of trial registry	9
Protocol	24	Where the full trial protocol can be accessed, if available	6
Funding	25	Sources of funding and other support (such as supply of drugs), role of funders	2, 20

*We strongly recommend reading this statement in conjunction with the CONSORT 2010 Explanation and Elaboration for important clarifications on all the items. If relevant, we also recommend reading CONSORT extensions for cluster randomised trials, non-inferiority and equivalence trials, non-pharmacological treatments, herbal interventions, and pragmatic trials. Additional extensions are forthcoming: for those and for up to date references relevant to this checklist, see www.consort-statement.org.

Table 2. **Checklist of Items To Include When Reporting Harms in Randomized, Controlled Trials***

Standard CONSORT Checklist: Paper Section and Topic	Standard CONSORT Checklist: Item Number	Descriptor	Reported on Page Number
Title and abstract	1	If the study collected data on harms and benefits, the title or abstract should so state.	1
Introduction			
Background	2	If the trial addresses both harms and benefits, the introduction should so state.	1
Methods			
Participants	3		
Interventions	4		
Objectives	5		
Outcomes	6	List addressed adverse events with definitions for each (with attention, when relevant, to grading, expected vs. unexpected events, reference to standardized and validated definitions, and description of new definitions).	8
		Clarify how harms-related information was collected (mode of data collection, timing, attribution methods, intensity of ascertainment, and harms-related monitoring and stopping rules, if pertinent).	7,9
Sample size	7		
Randomization			
Sequence generation	8		
Allocation concealment	9		
Implementation	10		
Blinding (masking)	11		
Statistical methods	12	Describe plans for presenting and analyzing information on harms (including coding, handling of recurrent events, specification of timing issues, handling of continuous measures, and any statistical analyses).	9
Results			
Participant flow	13	Describe for each arm the participant withdrawals that are due to harms and their experiences with the allocated treatment.	Figure 1
Recruitment	14		
Baseline data	15		
Numbers analyzed	16	Provide the denominators for analyses on harms.	10-12
Outcomes and estimation	17	Present the absolute risk per arm and per adverse event type, grade, and seriousness, and present appropriate metrics for recurrent events, continuous variables, and scale variables, whenever pertinent.†	Table 2
Ancillary analyses	18		
Adverse events	19	Describe any subgroup analyses and exploratory analyses for harms.†	Supp Table 1
Discussion			13
Interpretation	20	Provide a balanced discussion of benefits and harms with emphasis on study limitations, generalizability, and other sources of information on harms.‡	12-15
Generalizability	21		
Overall evidence	22		

* This proposed extension for harms includes 10 recommendations that correspond to the original CONSORT checklist.
† Descriptors refer to items 17, 18, and 19.
‡ Descriptor refers to items 20, 21, and 22.

Copyright ©2015 American College of Physicians
